# Enhancement of TiO_2_ NPs Activity by Fe_3_O_4_ Nano-Seeds for Removal of Organic Pollutants in Water

**DOI:** 10.3390/ma9090771

**Published:** 2016-09-10

**Authors:** Silvia Villa, Valentina Caratto, Federico Locardi, Stefano Alberti, Michela Sturini, Andrea Speltini, Federica Maraschi, Fabio Canepa, Maurizio Ferretti

**Affiliations:** 1 Department of Chemistry and Industrial Chemistry, University of Genoa, Genoa 16146, Italy; silvia.villa@chimica.unige.it (S.V.); federico.locardi@unige.it (F.L.); stefanofilippo.alberti@gmail.com (S.A.); fabio.canepa@unige.it (F.C.); ferretti@chimica.unige.it (M.F.); 2 Department of Chemistry, University of Pavia, Pavia 27100, Italy; michela.sturini@unipv.it (M.S.); andrea.speltini@unipv.it (A.S.); federica.maraschi@unipv.it (F.M.)

**Keywords:** titania, photocatalysis, methylene blue, Ofloxacin, fluoroquinolone

## Abstract

The enhancement of the photocatalytic activity of TiO_2_ nanoparticles (NPs), synthesized in the presence of a very small amount of magnetite (Fe_3_O_4_) nanoparticles, is here presented and discussed. From X-ray diffraction (XRD) and differential scanning calorimetry (DSC) analyses, the crystallinity of TiO_2_ nanoparticles (NPs) seems to be affected by Fe_3_O_4_, acting as nano-seeds to improve the tetragonal TiO_2_ anatase structure with respect to the amorphous one. Photocatalytic activity data, i.e., the degradation of methylene blue and the Ofloxacin fluoroquinolone emerging pollutant, give evidence that the increased crystalline structure of the NPs, even if correlated to a reduced surface to mass ratio (with respect to commercial TiO_2_ NPs), enhances the performance of this type of catalyst. The achievement of a relatively well-defined crystal structure at low temperatures (*T*_max_ = 150 °C), preventing the sintering of the TiO_2_ NPs and, thus, preserving the high density of active sites, seems to be the keystone to understand the obtained results.

## 1. Introduction

Nowadays, titanium dioxide is one of the most studied photocatalysts due to its low cost, versatility of synthesis, high chemical stability and, finally, high efficiency [1,2]. The fields in which TiO_2_ is applicable are widely diffuse, ranging from self-cleaning surfaces [3] to sterilization [4], photoelectrochemical conversion [5], clean energy production [6], and also air and water purification systems. In this last field, water treatment for the removal of organic (potentially) toxic substances [7,8], included pharmaceutically-active emerging pollutants [9,10], is currently a priority task. In fact, the material is able to produce, under ultraviolet (UV) excitation, electron-hole pairs that induce the formation of highly reactive species able to oxidize the organic matter [11]. Unfortunately, the UV component of the natural solar light irradiation spectrum (the highest and cheapest illumination source) is quite small; moreover, titanium dioxide is not able to absorb the solar radiation in the visible region. Therefore, the scientific and technological research in this field is strongly involved in find new possibilities to enhance the overall efficiency. Two different strategies can be generically followed. The first approach implies the use of opportunely doped ions able to shift the absorbed band gap from the UV to the visible: in this model some transition metals, such as Fe, V, and Zn, have been widely tested and adopted [12]. The second method is related to the increase of the intrinsic efficiency of the material through the control of the synthesis process; in this way it is possible to govern the surface area, the dimensions, and morphology, and, consequently, the photocatalytic activity. Further innovative systems have been recently developed, e.g., supporting the photocatalyst on the surface of luminescent materials, allowing an excellent increase in the degrading power [13].

In this work, we have studied the synthesis of nanoparticles (NPs) of titanium dioxide in the presence of magnetite nanoparticles. Fe_3_O_4_ NPs, presenting a superparamagnetic behavior below a typical critical dimension [14], have been evaluated as potential magnetic cores for Fe_3_O_4_@TiO_2_ hybrid material in order to facilitate the recovery (and recycling) of the catalyst applying an external magnetic field [15,16,17,18,19,20,21]. Generally, a thick SiO_2_ layer is inserted between the internal core (Fe_3_O_4_) and the external catalyst (TiO_2_) to prevent the Fe_3_O_4_-TiO_2_ direct contact that would decrease the photodegradation activity [22,23,24,25]. Indeed, it has been demonstrated that the TiO_2_ surface area decreased if supported on a magnetic substrate [26]. Consequently, our attention was focused to the possibility of using, in the titania sol-gel synthesis route, very small amounts of magnetite, exploited as germination seeds affecting the nucleation, formation, and morphology of the TiO_2_ NPs. Different syntheses have been carried out, varying the TiO_2_/Fe_3_O_4_ ratio. The photocatalytic performance has been tested in the framework of a standard ISO 10678:2010 protocol, i.e., the degradation of methylene blue (MB) solution of known concentration [27]. The results, hereafter reported, showed a strict correlation between the amount of Fe_3_O_4_ added during the TiO_2_ synthetic process and the observed titania photocatalytic activity; moreover, other properties, such as surface area and crystallinity, have been widely affected. Furthermore, a comparison of the photocatalytic activity of our NPs with a commercial product (TiO_2_ NPs P25 from Sigma-Aldrich, St. Louis, MO, USA), demonstrated the higher activity possessed by our materials. Consequently, the best material was also tested for the degradation of Ofloxacin (OFL), an important emerging water pollutant, belonging to the class of fluoroquinolone (FQ) antibiotics.

## 2. Results

### 2.1. Structural and Morphological Investigations

**Figure 1:**
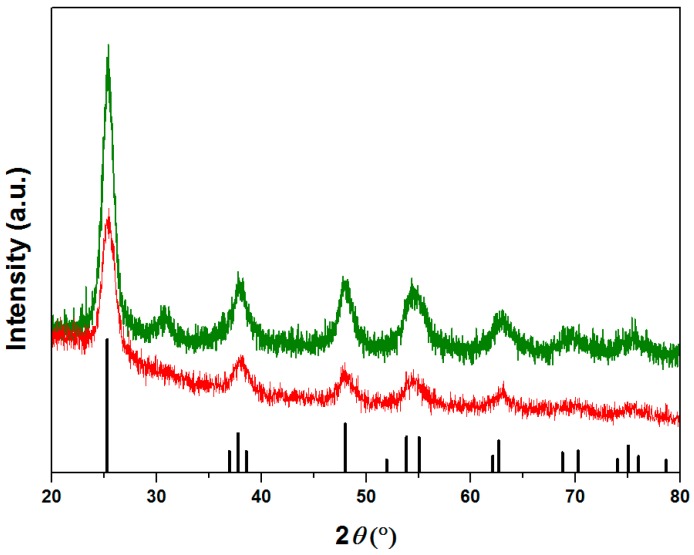
XRD patterns of Samples A (green curve), sample B (red curve), and crystallographic peaks of anatase phase (black line pattern). (Color figure online).

In Figure 1 the X-ray diffraction patterns of samples A and B are reported. The two compounds showed the characteristic peaks of the TiO_2_ tetragonal anatase structure, presented as a line pattern in the same figure. On the contrary, sample C and D (not reported in the figure) resulted completely amorphous and no identifiable peaks were detected. It is noteworthy that magnetite is well below the detection limit of the XRD technique due to its very low amount respect to the TiO_2_ matrix. It should be pointed out that sample A resulted more crystalline than sample B: this fact is underlined by the presence, in sample A, of higher intensity peaks and by a diffraction pattern better discernable from the baseline, especially at 2θ > 60°.

**Figure 2:**
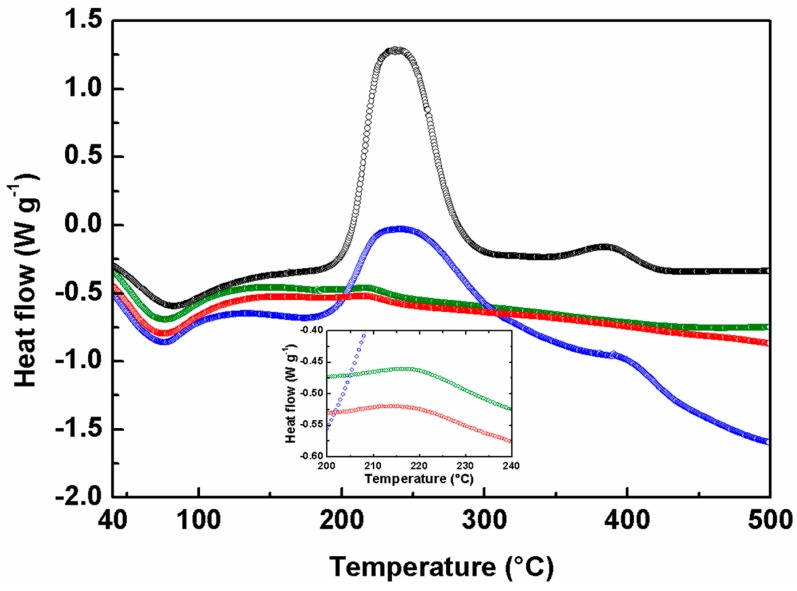
DSC analyses of sample A (green), sample B (red), sample C (blue) and sample D (black). In the inset the peaks at 215 °C for samples A and B are enlarged. (Color figure online).

These diffraction data are consistent with the DSC investigations, reported in Figure 2. The thermal behavior of all the samples is characterized by a broad endothermic peak below 100 °C, characteristic of the dehydration caused by a small quantity of water adsorbed. A second exothermic peak is observed at 215 °C for samples A and B and 240 °C for C and D, respectively.

In this step the oxidation of the 2-propanol adsorbed on the surface occurred [28]. The drastic difference in the intensity signals indicates a different amount of solvent that increased from sample A to D. Indeed, this result agree well with the surface area analysis from BET isotherms (Table 1), where the increased value of the surface to mass ratio from A to D, implies an increment of the possibility to adsorb the solvent. Samples A and B had no supplementary signals, demonstrating a complete crystallization already at 150 °C, a temperature notably lower with respect to the literature data [1,26]. On the contrary, samples C and D possessed weak exothermic peaks at 385 °C, correlated with the phase transition of the particles from amorphous to the tetragonal anatase structure. Consequently, the DSC investigation confirmed the data obtained from the XRD analysis, demonstrating the crystallization in the tetragonal anatase phase only for samples A and B.

**Figure 3:**
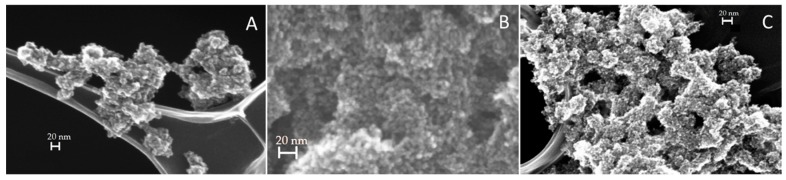
FE-SEM images of the three samples respectively prepared with (**A**) 0.71 mL; (**B**) 0.35 mL and (**C**) 0.18 mL of Fe_3_O_4_ NPs.

Figure 3 reports FE-SEM images of the three different magnetite-TiO_2_ samples. Apparently, no macroscopic differences were detected: all of the materials presented a very similar, roughly spherical, morphology with large aggregates in which the TiO_2_ NPs have dimensions of about 10 nm.

Finally, the surface area analysis, reported in Table 1, showed an evident increase in the surface to mass ratio, from 174.83–302.39 m^2^/g from sample A to sample C respectively. All of these values were lower than the pure TiO_2_ NPs surface area (D system with 341.86 m^2^/g). The increase in the surface area is strictly correlated to the decrease of the crystallinity grade, as the aforementioned XRD analysis revealed. Since the adopted synthesis protocol in all of the samples is the same, except the concentration of magnetite NPs, a clear dependence between the amount of Fe_3_O_4_ and the morphological and crystalline properties of the materials was observed. Decreasing the amount of magnetite, from sample A to D, a contemporary decrease in crystallinity and increase in surface area is clearly demonstrated. This general behavior can be correlated to the role of Fe_3_O_4_ nanoparticles that act, in the whole synthesis process, only as germination seeds for the growing and crystallization of TiO_2_ in the anatase phase.

### 2.2. Photocatalytic Activity

**Figure 4:**
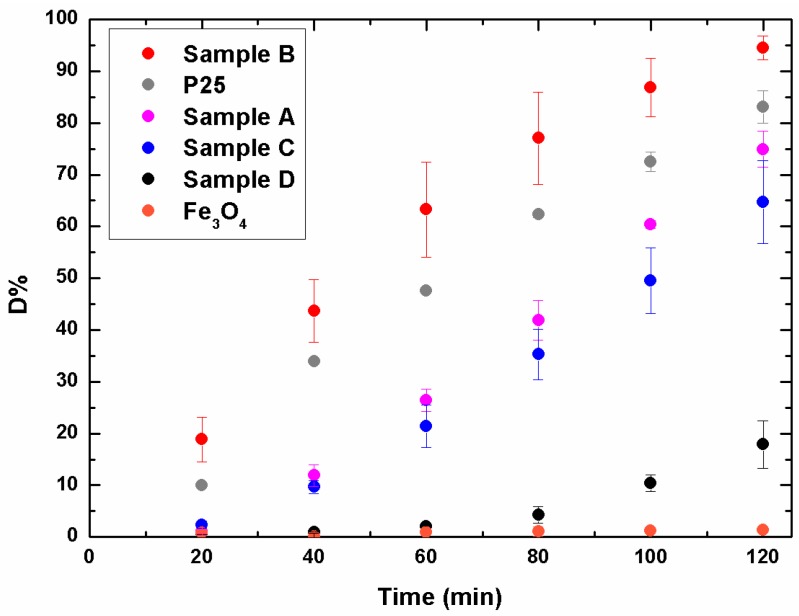
Percent degradation of MB versus time. The experimental points (Table S1) are averaged over three replicates. (Color figure online).

Figure 4 reports the efficiency of the methylene blue degradation using the hybrid TiO_2_/Fe_3_O_4_ samples (A, B, C) in comparison with pure TiO_2_ NPs (D), synthesized under the same conditions, but without the presence of magnetite, and commercial P25, i.e., TiO_2_ powders from Sigma Aldrich. Magnetite NPs, tested under the same conditions, do not show photocatalytic activity towards MB. Sample D results the less active even if presenting the best surface area; this fact is clearly related to the low crystallinity that drastically affects the catalytic performance [29]. Even if from the XRD result of sample C was also amorphous, its degradation efficiency is similar to sample A; apparently, the synergic effect between surface area and crystallinity played an important role. Consequently, sample A possessed a larger number of particles in the anatase structure, but suffered for the lack in surface area; on the contrary, for sample C the high surface area compensated the low crystalline grade. However, the two samples reach a degradation of about 70% after 120 min of light exposure, value lower of only 10% respect to P25. The best catalytic performance is reached by sample B, which shows a conversion up to 95% after 120 min. It is noteworthy that, already after 60 min, sample B degrades more than 60% of MB.

Finally, the photocatalytic efficiency of the best material was tested for the degradation of OFL, a real emerging contaminant, in natural water samples. Among FQs, this compound was selected due to its wide presence in environmental matrices [30], and also because it is characterized by a slower photolytic decay in water matrices compared to other drugs [31].

**Figure 5:**
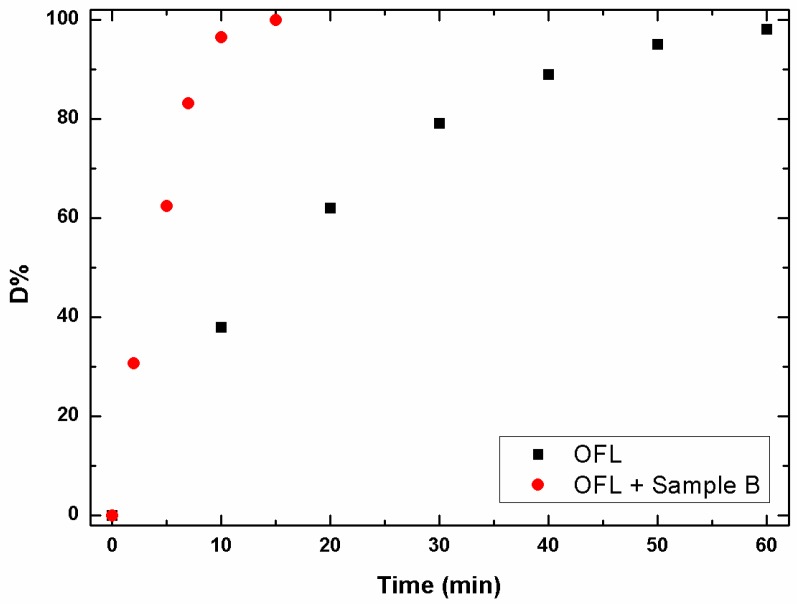
Photolytic (■) and photocatalytic (●) degradation profiles of OFL under simulated solar light. The experimental points (Table S2) are averaged over three replicates (RSDs < 5%, *n* = 3). (Color figure online).

Before irradiation, spiked samples (10 mg/L OFL, 0.5 g/L catalyst) were stirred in the dark, for 20 min to achieve sorption equilibrium. Under these conditions, a significant percentage of OFL was adsorbed onto the catalyst, namely 17%. As shown in Figure 5, a quantitative abatement (>96%) of OFL was gained under simulated solar light in just 10 min, with good reproducibility (RSD < 5%, *n* = 3). On the contrary, under the same experimental conditions, about 60 min were required to obtain a comparable degradation efficiency under direct photolysis. A non-exponential data decay was observed in both cases and the kinetic constants were 0.27(1) and 0.052(2) min^−1^, respectively.

## 3. Materials and Methods

### 3.1. Materials

FeCl_3_·6H_2_O (98%) and FeCl_2_·4H_2_O (98%), sodium hydroxide (97%), titanium isopropoxide (TISOP, 97%), 2-propanol (99.9%), and analytical grade Oflaxacin were all purchased from Sigma-Aldrich (St. Louis, MO, USA) and used as received.

HPLC grade acetonitrile (ACN) was from VWR (Radnor, PA, USA) and H_3_PO_4_ (85%, *w*/*w*) from Carlo Erba Reagents (Milano, Italy).

### 3.2. Preparation of Magnetite NPs

Magnetite nanoparticles were obtained by a coprecipitation method from an aqueous solution of stoichiometric amounts of FeCl_2_·4H_2_O and FeCl_3_·6H_2_O under basic conditions. Details in the preparation and the physicochemical properties of the sample are reported elsewhere [14]. Briefly, FeCl_2_·4H_2_O (2.5 mmol) and FeCl_3_·6H_2_O (5 mmol) were dissolved in Milli-Q water at pH 2 under N_2_ atmosphere and vigorous mechanical stirring. Once the solution reached 75 °C, a proper amount of NaOH aqueous solution (2 M) was quickly added, causing a sudden appearance of a black color in the solution. The reaction was continued for 30 min, after which the particles were washed several times with boiling water and magnetically collected after each wash, in order to reach neutral pH. Finally, a known volume of water was added to disperse ultrafine magnetic particles to a final concentration of 17 g/L.

### 3.3. Preparation of Magnetic Photocatalysts

Different amounts of Fe_3_O_4_ NPs were used in this step (Table 2). At first, a given volume of the Fe_3_O_4_ suspension was diluted in 10 mL of 2-propanol and sonicated for 5 min at 30% power with an OMNI Sonic Ruptor Ultrasonic Homogenizer (Omni International, Atlanta, GA, USA). Then the dispersion was transferred to 200 mL of propanol and a given amount of TISOP was added. Next, 30 mL of water, as reported in Table 2, were added, producing the immediate formation of TiO_2_ nanoparticles. The reaction proceeds at room temperature for 4 h after which the dispersion undergoes a hydrothermal process at 150 °C for 3 h.

For each A, B, C, and D system, two different samples were prepared and fully characterized.

### 3.4. Characterization Techniques

#### 3.4.1. XRD

X-ray diffraction patterns on dried NPs were performed on a Philips PW1830 diffractometer (Philips, Amsterdam, The Netherlands) using the Bragg–Brentano geometry, with Cu Kα radiation (λ = 0.15406 nm), Ni filtered. The data were collected in the 20°–80° 2θ range, with a step of 0.025° and a counting time for each step of 5 s.

#### 3.4.2. FE-SEM

FE-SEM (Field Emission–Scanning Electron Microscopy) analyses were performed using a ZEISS SUPRA 40VP microscope (Carl Zeiss AG, Oberkochen, Germany) equipped with an energy dispersive X-ray spectrometer (EDXS OXFORD “INCA Energie 450x3”, Oxford Instrument, Abingdon, UK) for microanalysis, using the low voltage (5 kV) mode. The analyses were performed collecting the signal by means of the In-Lens detector.

#### 3.4.3. DSC

Differential scanning calorimetry (DSC) measurements were carried out through a Mettler Toledo 821e calorimeter (Mettler Toledo, Columbus, OH, USA) in O_2_ atmosphere (20 mL/min), using aluminum crucibles. The data were collected in the 40–500 °C temperature range with a heating rate of 10 °C/min.

#### 3.4.4. BET

BET surface areas of the samples were obtained from nitrogen adsorption isotherms acquired using an ASAP 2010 physisorption analyzer (Micromeritics Instrument Corp., Norcross, GA, USA). Before the measurement each sample was pre-treated at 200 °C in vacuum for 12 h.

### 3.5. Photocatalytic Experiments

The photocatalytic activity was tested using, for each sample, 12.5 mg in 25 mL of an aqueous methylene blue (MB) solution (0.04 g/L). Before the irradiation, the suspensions were kept in the dark under magnetic stirring for 20 min to establish the adsorption-desorption equilibrium between the particles and the dye. The suspensions were then exposed to a solar spectrum lamp 300W Ultra-Vitalux (Osram, Munich, Germany) maintained at 20 cm distance for 2 h at room temperature. Aliquots of samples (1.5 mL) were taken before irradiation (*t*_0_) and every 20 min until the end of the experiment. The collected samples were centrifuged by a Centrifuge 5410 (Eppendorf, Hamburg, Germany) at 13,200 rpm for 5 min to separate the NPs and the solution that was analyzed by means of a UV-VIS spectrometer (Lambda 35, Perkin Elmer, Waltham, MA, USA). The MB concentration was calculated monitoring the absorbance at 664 nm [11].

Irradiation of OFL aqueous solutions was performed by using a solar simulator Solar box 1500e (CO.FO.ME.GRA, Bologna, Italy) set at a power factor of 500 W/m^2^, equipped with a UV outdoor filter of soda lime glass, IR treated. A 100 mL tap water sample from the municipal waterworks of Pavia (pH 7.7, conductivity at 20 °C 271 μS·cm^−1^) spiked with 10 mg/L OFL was irradiated in a closed glass container (40 mm depth, exposed surface 9500 mm^2^). The catalyst suspension (0.5 g/L) was magnetically stirred in the dark for 20 min to promote the antibiotic adsorption on the catalyst surface. During irradiation, aliquots (1 mL) of each sample were withdrawn at the specific times (see Figure 5), filtered (0.2 µm) and promptly injected in the HPLC-UV system (LC-20AT solvent delivery module equipped with a DGU-20A3 degasser and interfaced with a SPD-20A UV detector (Shimadzu, Milano, Italy). The analysis wavelength selected was 275 nm. Twenty microlitres of each sample was injected into a 250 × 4.6 mm, 5 µm Analytical Ascentis C18 (Supelco, Sigma Aldrich Corporation, Milano, Italy) coupled with a similar guard column. The mobile phase was 25 mM H_3_PO_4_^−^ACN (85:15), at a flow rate of 1 mL/min. The instrumental quantification limit was 0.06 mg/L.

The percentage of degradation (D%) was determined using the relation [32,33]:

D% = (C_0_ − C_t_/C_0_) × 100

## 4. Conclusions

In this study, a simple and facile sol-gel process approach was developed for the preparation of Fe_3_O_4_—TiO_2_ nanopowders. Different molar ratios have been studied to test their photocatalytic activity in the degradation of MB dye and OFL antibiotic, an emerging water pollutant, in the UV–visible light range.

Fe_3_O_4_ NPs can improve the photocatalytic activity of titania due to the promotion of crystallization of the anatase structure at a lower temperature than that reported in the literature, since they can act as germination seeds. The combination of higher crystallinity grade and surface area provides an enhancement in the photocatalytic activity for sample B: the efficiency of our NPs in the MB and OFL degradation is even higher than the P25 commercial titania powders. Indeed, the percentage degradation reached, with the same exposure time, is 15% greater compared to commercial P25 TiO_2_.

## Figures and Tables

**Table 1:** Surface areas determined for the different samples.

Sample	BET Surface Area (m^2^/g)
A	174.83
B	286.73
C	302.39
D	341.86

**Table 2:** Reagents ratios (*v*/*v*) for the different samples.

Sample	Fe_3_O_4_ NPs (mL)	TISOP (mL)	2-Propanol (mL)	H_2_O (mL)
A	0.71	6	200	30
B	0.35	6	200	30
C	0.18	6	200	30
D	0	6	200	30

## Supplementary Materials

The following are available online at www.mdpi.com/1996-1944/9/9/771/s1.
