# Supplementary material for: Enhancement of TiO2 NPs Activity by Fe3O4 Nano-Seeds for Removal of Organic Pollutants in Water
Source: Materials (Basel). 2016 Sep 10;9(9):771. doi: 10.3390/ma9090771 (PMC5457040; doi:10.3390/ma9090771)
Supplement: Supplementary file 1 [file materials-09-00771-s001.pdf]

Supplementary Materials: Enhancement of TiO_2 NPs Activity by Fe_3O_4 Nano-Seeds for Removal of Organic Pollutants in Water

Silvia Villa, Valentina Caratto, Federico Locardi, Stefano Alberti, Michela Sturini, Andrea Speltini, Federica Maraschi, Fabio Canepa and Maurizio Ferretti

Table S1. Percent degradation of MB for the different materials. The experimental values are averaged over three replicates.

	Time (min)	20	40	60	80	100	120
**Sample A**	D%	1.1	11.9	26.4	41.9	60.4	74.9
	SD	0.5	2.1	2.2	3.9	0.7	3.5
**Sample B**	D%	18.9	43.7	63.3	77.0	86.9	94.6
	SD	4.3	6.0	9.2	8.9	5.7	2.3
**Sample C**	D%	2.4	9.7	21.4	35.3	49.5	64.7
	SD	0.7	1.3	4.1	4.8	6.3	8.0
**Sample D**	D%	0.6	0.9	2.1	4.3	10.4	17.9
	SD	0.2	0.1	<0.1	1.6	1.6	4.6
**P25**	D%	10.0	34.0	47.6	62.3	72.6	83.1
	SD	0.5	0.6	0.5	0.6	1.9	3.2
**Fe_3O_4**	D%	0.8	0.1	0.9	1.1	1.2	1.4
	SD	<0.1	0.8	0.1	0.9	<0.1	<0.1

Table S2. OFL percentage concentration under photolytic (OFL) and photocatalytic (OFL + Cat.) degradation under simulated solar light. The experimental points are averaged over three replicates. The D% values in Figure 5 are calculated according to the equation in 3.5 paragraph.

**OFL**			**OFL + Cat.**		
**Time (min)**	**OFL%**	**SD**	**Time (min)**	**OFL%**	**SD**
10	62.3	2.0	2	69.3	3.0
20	38.4	1.8	5	37.5	0.1
30	21.0	0.5	7	16.9	0.6
40	10.8	0.5	10	3.6	0.1
50	4.8	0.1	15	0	<0.1
60	1.5	<0.1			
